# Evaluation of knowledge, attitude, practices and effectiveness of menstrual hygiene interventions in rural schools from Lilongwe, Malawi

**DOI:** 10.1186/s12889-024-18940-w

**Published:** 2024-05-29

**Authors:** Russel Chidya, Olivia Kachuma, Tchaka Thole, Louis Banda, Mark Loewenberger, Jennifer Nicholson

**Affiliations:** 1 Department of Water and Sanitation, Mzuzu University, P/Bag 201. Luwinga, Mzuzu, Malawi; 2 Innovation Research and Training Centre (INNORET), Head Office, P.O. Box 195, Mzuzu, Malawi; 3 Canadian Physicians for Aid & Relief (CPAR), P.O. Box 30998, Lilongwe, Malawi

**Keywords:** Education, Lilongwe, Menstrual hygiene management, Menstrual health and hygiene, Water sanitation and hygiene

## Abstract

**Background:**

Menstrual hygiene management (MHM) is associated with the menstrual process in women and adolescent girls who face cultural and financial challenges in rural areas of many developing countries. As part of the pilot study, we assessed the sustainability and effectiveness of the approaches and lessons learned from the MHM project intervention in rural areas of Lilongwe, Malawi.

**Methods:**

Rural primary schools (*n* = 4) were purposively selected where an MHM intervention was implemented in Lilongwe, Malawi. The study employed a mixed-method research design. Assessments and data collection were performed through surveys of learners, literature reviews, key informant interviews (KIIs) (*n* = 90), and 20 focus group discussions (FGDs). The study participants included boys and adolescent girls (*n* = 100, 11–19 years; grades 5–8), teachers, mother groups, and community leaders from the selected schools.

**Results:**

All the schools had water sanitation and hygiene facilities and latrines (45% improved, 54% ventilated improved pit latrines – VIPs) that promoted menstrual hygiene for adolescent girls. However, two of the schools studied (50%, *n* = 4) did not have separate washrooms for changing sanitary materials. There was a slight increase in latrine coverage in Kabuthu zone communities (90% at baseline versus 93.4% at midterm). However, the coverage dropped to 85.7% at the final evaluation, which was attributed to too much rain received in the area that damaged most of the latrines. There was a significant reduction (*p* < 0.05) in the number of girls failing to attend classes due to menstruation (70% at baseline versus 14% at final evaluation). Furthermore, the project resulted in the majority of girls (94.4%) having access to school. There was a strong uptake and adoption of sanitary products (reusable pads and menstrual cups) among adolescent girls of all age groups. The study has demonstrated that the inclusion of key stakeholders such as health workers, parents, mother groups and community leaders promoted the uptake and sustainability of reusable pads and menstrual cups and MHM interventions and programs.

**Conclusion:**

The MHM project implementation improved adolescent girls’ education in the area. The inclusion of boys and other key stakeholders in the health education talks addressed issues of stigma and discrimination. The study, therefore, calls for comprehensive training on MHM and hygiene education to remove discrimination and harmful cultural practices.

**Supplementary Information:**

The online version contains supplementary material available at 10.1186/s12889-024-18940-w.

## Introduction

### Background information

Women and girls, especially from developing countries, are faced with problems in managing their menstruation as a result of cultural taboos, lack of knowledge, inadequate access to safe and secure water, sanitation, hygiene (WASH) services, and lack of affordable menstrual products [1, 2]. According to WHO/UNICEF, menstrual hygiene management (MHM) is simply defined as the management of hygiene associated with the menstrual process. Furthermore, adequate MHM is ‘*access to clean absorbents including sufficient washing, drying, storage and wrapping of reusable absorbents; adequate frequency of absorbent change; washing the body with soap and water; adequate disposal facilities; privacy for managing menstruation; and basic understanding of menstruation and how to manage it with dignity and without fear or embarrassment’* [1, 3]. The concept of MHM has been expanded to menstrual health and hygiene (MHH) to encompass the sociocultural and economic elements that influence the menstrual management of women and girls [3, 4].

The provision of adequate MHH among girls and women is reported to contribute to the attainment of Sustainable Development Goals (SDGs) for good health (SDG 3), education (SDG 4), gender equality (SDG 5), and clean water and sanitation (SDG 6) [3]. However, many girls and women in low- and middle-income countries (LMICs) do not have adequate MHH services [3, 4]. Access to WASH services for MHM and MHH must be considered a basic right of girls and women to lessen gender discrepancies in education, health and sociopolitical and economic participation [4, 5]. Previous studies in Sub-Saharan Africa and elsewhere have shown that WASH interventions through the provision of sanitary materials, water, soap and privacy keep girls in school and have a mixed impact on school absenteeism and performance [6–8]. In Malawi, various institutional/legal frameworks and strategies on gender equality, the empowerment of women and girls and human dignity are employed by several key stakeholders and organizations. For example, the Canadian Physicians for Aid & Relief (CPAR) Malawi implemented an MHM project intervention in Lilongwe rural schools aligned with Global Affairs Canada’s Feminist International Assistance Policy (FIAP). Furthermore, the MHM project implementation is in line with the National Gender Policy (2015), which aims to increase advocacy for girls’ and boys’ conducive learning environment, and Malawi 2063, which stresses the need to reinforce the gender equality and empowerment of women and girls to shape their decisions at the household, community and national levels.

Malawi has approximately 50.7% of its population living below the poverty line and approximately 25% living in poverty, especially in rural areas [3]. Poverty is exacerbated by low educational outcomes, including failure to complete primary schooling, especially among girls and women in rural areas. As girls start menses, they begin to miss school activities to the point where they drop out of school entirely, jeopardizing their ability to contribute more effectively to their own and their families’ social and economic well-being. A study conducted under the Malawi Red Cross showed that girls in their menses had a significantly higher level of knowledge compared to boys, and knowledge in girls was associated with better MHM practices and with reduced absenteeism [9]. A study conducted in seven schools in and around Lilongwe, Malawi by WaterAid in 2021 concluded 3 recurring issues impacting MHM for adolescent girls: (a) cultural around menstruation – menstruation being seen as strictly secret, and parents do not talk to children about it; (b) ignorance about menstrual issues being prevalent among schoolgirls and in their communities; and (c) inadequate WASH facilities and infrastructure in visited schools. Several projects and interventions on MHM and MHH have been implemented in rural Malawi to address such issues and challenges [8–10].

### Context of the study

Several studies on linkages between menstruation and school absenteeism have been widely conducted in Africa and elsewhere [8, 11–13]. An earlier study by Grant et al. [14] on menstruation and absenteeism in rural Malawi noted several factors contributing to menstruation-related absenteeism. This included lack of school toilet privacy, lack of education by family and teachers on puberty and MHM, and physical menstrual discomfort. In the same study, long distances between home and school also prevented girls from going back and forth, hence contributing to absenteeism [14]. Such barriers and challenges significantly disrupt girls’ education and development, which later leads to ripple effects. There are no recent disaggregated data on school drop-out rates in Malawi [9]. However, UNICEF Malawi gathered significant anecdotal evidence that girls stay home while menstruating [10].

Menstrual cup promotion has been promoted among university girls at Mzuzu University in Malawi [10]. Although the project was successful, a small sample size (10 women) was used. Furthermore, the project did not target girls below university age, and there was no follow-up program. Many MHM projects in Malawi have focused simply on providing menstrual products and some WASH activities [8–10]. This addresses immediate short-term needs but does not incorporate the approach needed to promote sustainable change. The CPAR MHM project implemented an innovative MHM project in rural Lilongwe through a participatory approach by testing and promoting the use of menstrual cups and reusable pads alongside MHM behavior change interventions. The current study on MHM and MHH, therefore, provides a significant opportunity for knowledge sharing among WASH practitioners, education, and health promoters in the country. This study, therefore, assessed the sustainability and effectiveness of the approaches and lessons learned from the CPAR MHM/MHH project intervention for scaling up and replicability in other rural areas in Malawi and elsewhere. It aimed to evaluate knowledge, attitudes, and practices among learners, teachers and community members and the effectiveness and sustainability of menstrual hygiene interventions in rural schools from Traditional Authority (TA) Kabudula in Lilongwe, Malawi. The study aimed to achieve the following specific objectives: *(a) to evaluate availability and access to water and sanitation facilities, menstrual products, and menstruation management among adolescent girls; (b) to assess the knowledge, attitude, and practices regarding MHM among learners (girls and boys), teachers and community members in the study area; (c) to assess the project intervention on feasibility, acceptability, and changes in menstruation-related knowledge, practices, perceptions, and self-reported school absenteeism from baseline to end line among girls; and (d) to determine the sustainability, replicability and scalability of the MHM interventions in rural schools from Malawi and elsewhere.*

### Theoretical framework of the study

This study is grounded on a general hypothesis that the inability of girls up to the age of 18 to effectively manage their menstrual health is a significant cause of absenteeism and school dropout and that the use of menstrual cups and/or reusable sanitary pads improves girls’ menstrual health management and reduces absenteeism and dropout rates. The implementation of the MHM project was centered on gender-based analysis (GBA). The GBA underpins an understanding that health variances between boys (or men) and girls (or women) can be related to the various roles and responsibilities that culture assigns to them. This study employed the “*Theory of Change*” (*ToC*) approach to evaluate the knowledge, attitudes, and practices among learners, teachers and community members and the effectiveness of menstrual hygiene interventions in rural schools from the study area. Furthermore, the study used the “*Theory of Planned Behavior*” (TPB) [15]. The *ToC* is a theory that gives a detailed description and illustration of how and why the desired change is expected to happen in a particular situation [16]. It explains how activities are understood to contribute to a series of results that produce the final intended impacts. The *TPB* depicts that human action is influenced by three major factors: a favorable or unfavorable evaluation of the behavior, perceived social pressure to perform or not perform the behavior, and perceived capability to perform the behavior [15].

## Methodology

### Study setting and MHM interventions

**Fig. 1:**
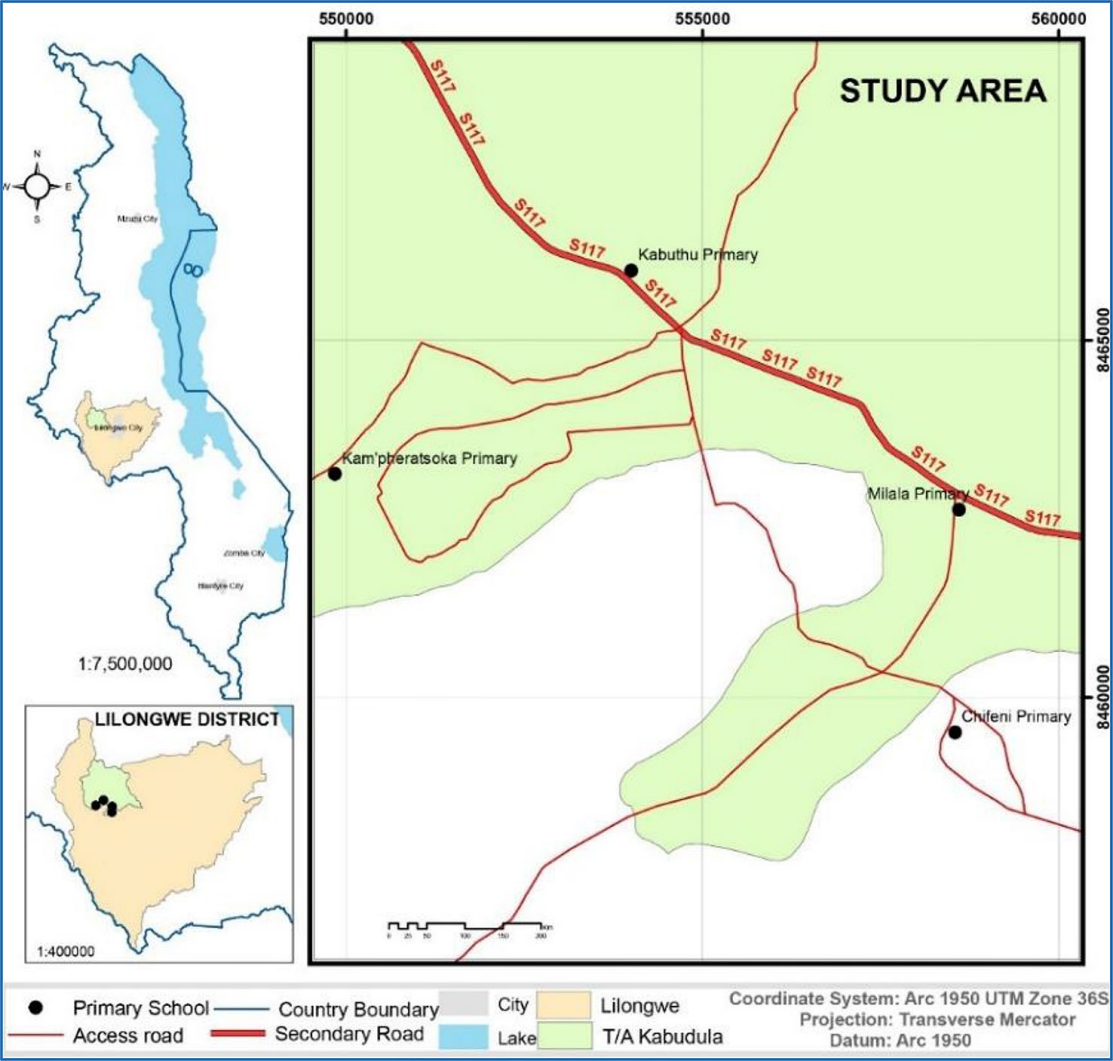
Map of Lilongwe showing the study area. *Source* Authors

This study was conducted in Lilongwe District located in the central region (Fig. 1). It is the capital city of Malawi, which stands at an altitude of 1,050 m. Specifically, the study was conducted in TA Kabudula under the Kabuthu education zone where rural primary schools (*n* = 4), namely, Kabuthu, Milala, Kamphelatsoka, and Chifeni, were selected. The schools under study were purposively selected based on the implementation of the MHM project by CPAR. The rural locations were initially identified by CPAR’s Rapid Gender Analysis (RGS) (September 2021) and more in-depth GBA (December 2021). The two analyses showed traditional and cultural beliefs being more ingrained, less available menstrual products and unaffordable, and having fewer opportunities to acquire information to dispel misconceptions and ignorance regarding MHM. The CPAR MHM/MHH project interventions implemented in the area included training and provision of menstrual cups and sanitary pads to adolescent girls from the four schools. Furthermore, the project involved MHM education, awareness campaigns and capacity building among adolescent boys, teachers, mother groups, girls’ councilors, traditional leaders, school and health management committees; parents and community members.

### Study design, methods, and data collection tools

In July and August 2023, we implemented a mixed-methods research design where both quantitative and qualitative data were collected. Quantitative assessment was performed through school and *household surveys.* Quantitative data were collected through surveys of learners in the 4 selected schools, while qualitative data were collected using direct observations, key informant interviews (KIIs) or in-depth interviews (IDIs) and focus group discussions (FGDs). A literature review of project documents (baseline, midterm, and end-line progress reports) was made. Furthermore, community-level knowledge-sharing workshop reports and other related MHM/MHH project documents were reviewed and analyzed. The girl’s MHM questionnaire, boys’ FGD guide, household MHM questionnaire, and mother group’s FGD guide were prepared and implemented.

### Participants, sample size and inclusion criteria

The study participants included adolescent boys and girls (11–19 years; grades 5–8), Teachers/Head Teachers, Mother Group members, Girls Councilors, Parent-Teacher Association (PTA), Chiefs/GVH, School Management Committees (SMCs), and Community Health Committees. We purposively selected and surveyed a total of 100 adolescents girls at the menarche stage to understand their level of knowledge, adoption, uptake and impact of the MHM interventions. Next, a total of 90 households where the adolescents came from were engaged in interviews to sort their understanding and support of the MHM to their adolescent girls. The study targeted households (HH) with adolescent girls only because of their experience in managing girls during menstruation. Due to the scarcity of such types of households, the study targeted the nearest HHs with such girls. In addition, 8 FGDs, 4 KIIs and 1 survey with boys on myths and misconceptions were conducted in each of the four schools. A total of 20 FGDs were conducted in all the schools, with groups of 5–10 people purposively selected for their first-hand information. Discussions were centered on selected topics specifically on WASH and menstruation while allowing for interesting, new, or unplanned follow-up questions to be asked. Concurrently, direct observation was used to confirm or triangulate the information given through the questionnaire survey and photographic pictures.

The sample size and distribution of participants are summarized and presented in Table 1. The study used convenience and purposive sampling to identify study participants, namely, teachers, mother group members, PTAs, chiefs, health center management groups, youth groups, and parents. Participants who had stayed in the area for at least 6 months and/or were involved in the MHM/MHH interventions were engaged. To validate the quantitative data, KIIs targeting key districts and community-level informants connected to adolescent girls were employed. Furthermore, the study participants were selected using the inclusion and exclusion criteria summarized in Table 2. Android tablets programmed in mWater were used for data collection to minimize errors since the program was developed with built-in data validation, skip rules, and constraints in the questionnaire.

**Table 1:** Summary of study participants, key informants and FGD sample size

Study Participant	Sample size				Subtotal
	Kabuthu	Milala	Kampheratsoka	Chifeni	
*Adolescent Girls*	25	25	25	25	100
*Adolescent Boys*	10	10	10	10	40
*Teachers/Head Teacher*	1	1	1	1	4
*Mother Group members*	1	1	1	1	4
*Girls Councilors*	1	1	1	1	4
*PTAs*	1	1	1	1	4
*Chiefs/GVH*	1	1	1	1	4
*School Management Committees*	1	1	1	1	4
*Community Health Committees*	1	1	1	1	4
*Households (parents and community members)*	22	22	23	23	90
*District Health Officer*	1				1
*District Water Officer*	1				1
*District Project manager*	1				1
Total study participants					261

PTA: Parents-Teachers Association. GVH: Group Village Heads

**Table 2:** Summary of study participants’ inclusion and exclusion criteria

Participant	Inclusion and exclusion criterion
*Girls*	Only adolescent and school-going girls (11–19 years) (grades 5–8) enrolled in the selected schools only
*Boys*	School-going boys enrolled in the selected schools only
*Teachers*	Only those employed in the selected and target school.
*Mother Group members*	Only those members who belong to the group, stay in the area and were involved in the implementation of the MHM interventions.
Parent-Teacher Association	Members who linked to the selected schools and stay in the area
*Chiefs*	Traditional or religious leaders in the target area.
*Health Center Management groups*	Working in a health center familiar with the cultural practices of the area and involved in the MHM interventions
*Youth groups*	Youth groups operating in the area and were involved in the MHM interventions
*Parents and community members*	Parents and members staying in the target area for more than 1 year.

### Data management and statistical analysis

The qualitative data were analyzed manually through content analysis to contextualize quantitative findings. First, the qualitative data were entered into Microsoft Excel and Word for transcription, translation and cleaning. Preliminary reading of all reports was performed to identify initial key issues. All three approaches to qualitative content analysis as outlined in [17] were employed. The study assessed the MHM/MHH project intervention uptake and impact by computing prevalence differences at 95% confidence intervals using fixed-effects logistic regression. To ensure the validity and reliability of the results, the authenticity of statements and information received were verified with experts and stakeholders in the area. Furthermore, all the data collection tools were piloted and pretested for their efficiency and correctness before the actual data collection.

### Ethical consideration

#### Ethical approval

To conduct this study, ethical approval was obtained from the Mzuzu University Research Ethics Committee (MZUNIREC) (Ref. No. MZUNIREC/DOR/23/95). During the implementation of the MHM/MHH interventions in the area, permission and partnerships were made with all relevant government offices, including Ministries of Health, Education, and Community Health Centers outreach clinics. Confidentiality was followed, and instead of names, identification codes were used for analysis purposes. A written informed consent form was signed or thumb printed before any form of data collection. For adolescent boys and girls under 18, written informed assent was obtained from their parents, guardians, or teachers. The adolescent boys and girls aged 18 and above provided consent to take part in the interventions and study.

## Results

### Access to water and sanitation facilities in schools and at home

#### In schools

The study findings showed that all schools had latrines (45% improved, and 54% ventilated improved pit latrines – VIPs, *n* = 4). Despite having separate latrines for boys and girls, all the schools lacked handwashing facilities. As an alternative, schools drew water using basins and buckets and provided it to the students by placing them in front of classrooms for easy access. Although water was available for the students, no soap was provided for cleaning and handwashing. During midterm evaluation, it was noted that the Milala school had no water source following the theft of a pump and vandalized boreholes. At this school, students reportedly carried some water in small bottles to cater to all basic sanitation needs. Only the Milala and Kampheratsoka schools had separate washrooms for changing sanitary materials for adolescent girls. Coupled with the unavailability of water and soap in the washrooms and latrines, the study noted that adolescent girls had challenges cleaning themselves at school. However, this circumstance did not pose a significant impact (*p* < 0.05) on school attendance among the girls, as depicted by improved school attendance during the reporting period.

#### In communities surrounding the schools

**Fig. 2:**
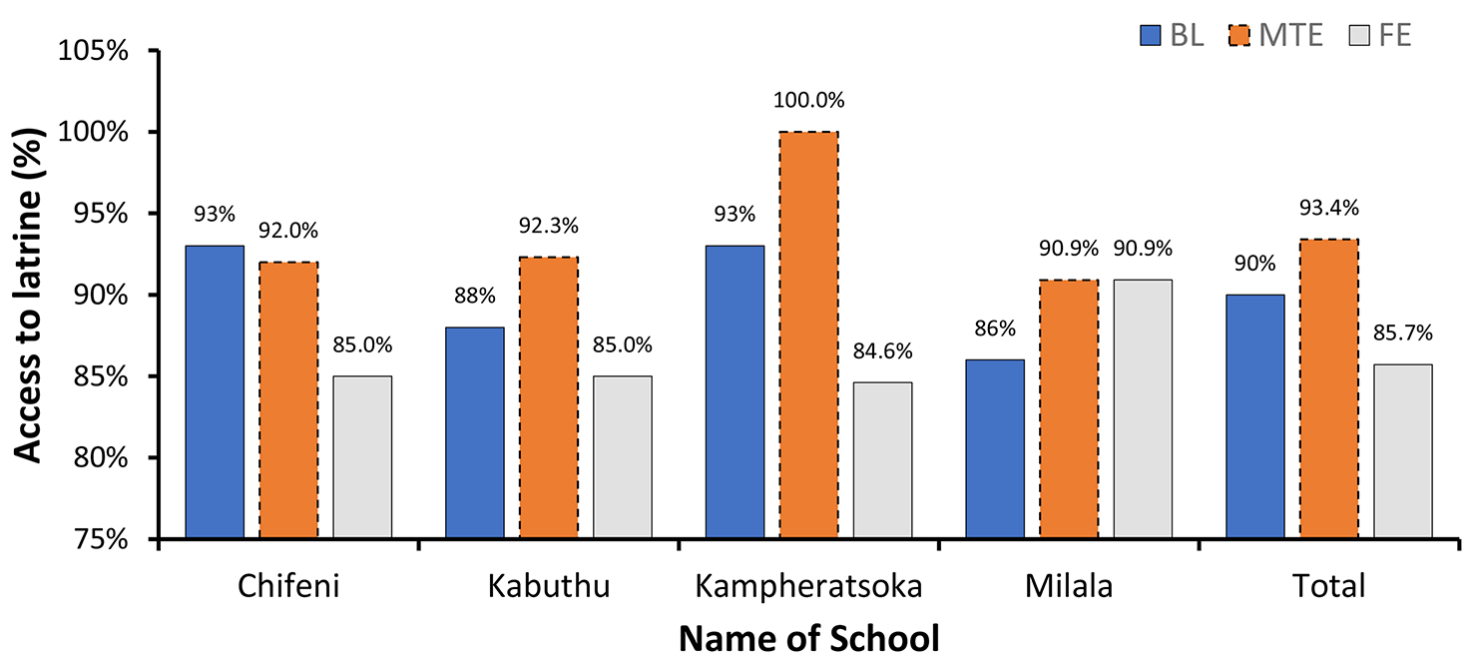
Access to latrines in the communities surrounding the schools in the study area

**Fig. 3:**
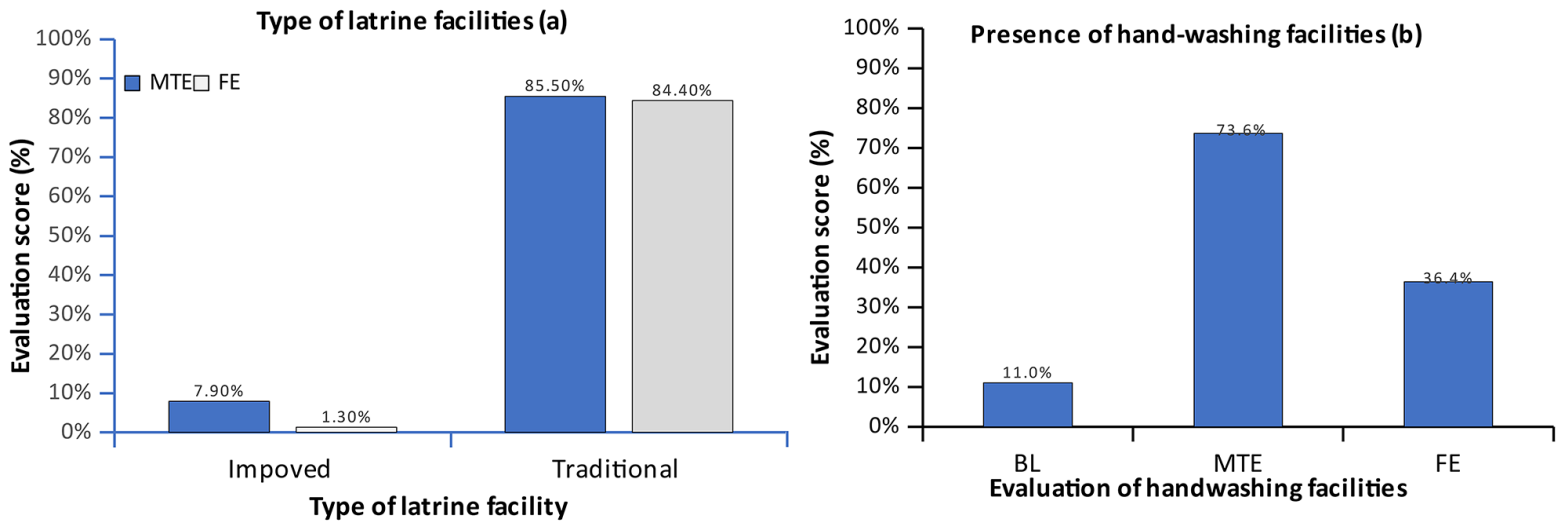
Type of latrine (**a**) and hand-washing facilities (**b**) used in surrounding communities

The results showed that there was a significant variation (*p* > 0.05) in access to latrines in the communities surrounding the schools (Fig. 2). There was a slight increase in latrine coverage in the Kabuthu zone (90% at baseline (BL) versus 93.4% at midterm evaluation (MTE), *n* = 4). However, the coverage dropped to 85.7% at the final evaluation (FE). During the final evaluation, the majority of people used traditional latrines (84.4%) (Fig. 3). Conversely, there was a moderate increase (11 to 36.4%) in the presence of hand-washing facilities in homes around the schools (Fig. 3). The increase was attributed to the engagement meetings where parents were urged to ensure girls had access to sanitation facilities at home. Improved access to water resulted in adolescent girls being able to clean up and maintain hygiene during menstruation.

### Absenteeism from primary school

#### Girls missing out on classes because of menstruation

The study showed a significant reduction (*p* < 0.05) in the number of girls failing to attend classes due to menstruation from 70% at baseline to 21% at midterm and 14% at the final evaluation. All the girls interviewed (100%, *n* = 90) felt comfortable using sanitary products, hence resulting in girls having more classroom learning time, similar to their male counterparts. Furthermore, the FGDs conducted with both teachers and parents revealed that there had been an improvement in the performance of girls during the project compared to the pre-project period. Despite helping reduce absenteeism, the improved availability of menstrual services in the area were noted to bring enormous positive impact and reduction in stigma and discrimination against girls.

#### Number of days girls missed classes due to menstruation

The number of girls who missed classes and stayed away from school due to monthly periods of less than 3 days, 3 to 5 days and more than 5 days decreased significantly. The girls who missed classes for less than 3 days because of monthly periods reduced from 18 to 8%, while for 3 to 5 days, there was a significant reduction from 63 to 6% (*p* < 0.05). For girls missing classes for more than 5 days, the number reduced to 0% because of the availability of sanitary pads and menstrual cups that helped them manage their menses well. The reduction in the number of days girls stay away from school has had a positive change in school academic performance comparable to that of boys, owing to the availability of MHM services and products.

### Improved access to schooling as a result of MHM support

#### Percentage of girls accessing improved education

**Fig. 4:**
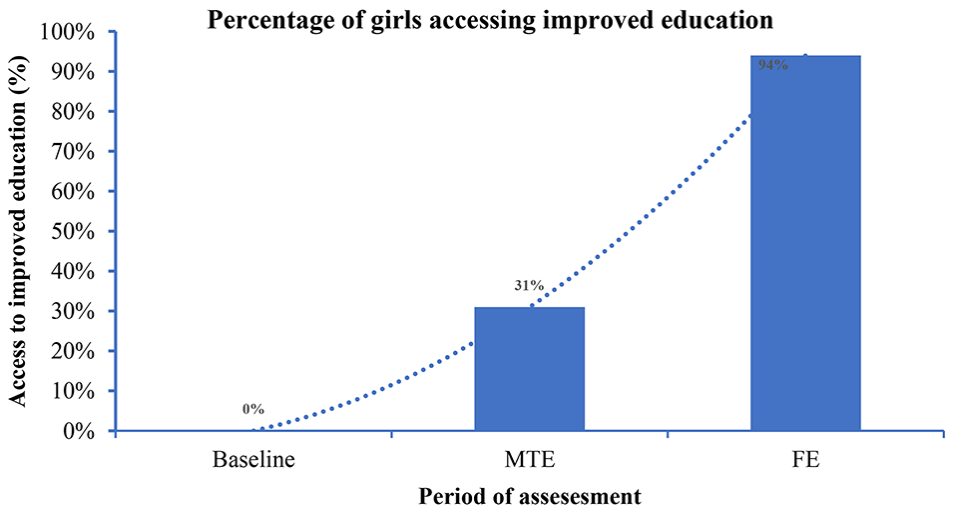
Percentage of girls accessing improved education after the project intervention

The majority of girls who participated in the program (94.4%, *n* = 100) reported improved access to school as a result of overall MHM support provided during the project implementation (Fig. 4). Many adolescent girls sampled (83.2%, *n* = 100) reported having used reusable sanitary materials compared to baseline findings where only 10% had access and used the same. Similarly, a considerable number of adolescent girls (70%) reportedly used menstrual cups. Thus, the increase in the percentage of girls accessing improved education was attributed to the availability and usage of sanitary pads and menstrual cups, hence resulting in reduced absenteeism. On average, each adolescent girl was reported to have a minimum of 5 reusable sanitary pads and 1 menstrual cup. In addition, the increase in the number of girls accessing improved education was attributed to awareness of menstrual hygiene among boys who stopped bullying girls when in menses. The awareness focused on myths and misconceptions related to MHM as well as the negative impacts of bullying that result in girls staying away from school or completely dropping out.

#### Number of learners comfortable attending school during menstruation

**Fig. 5:**
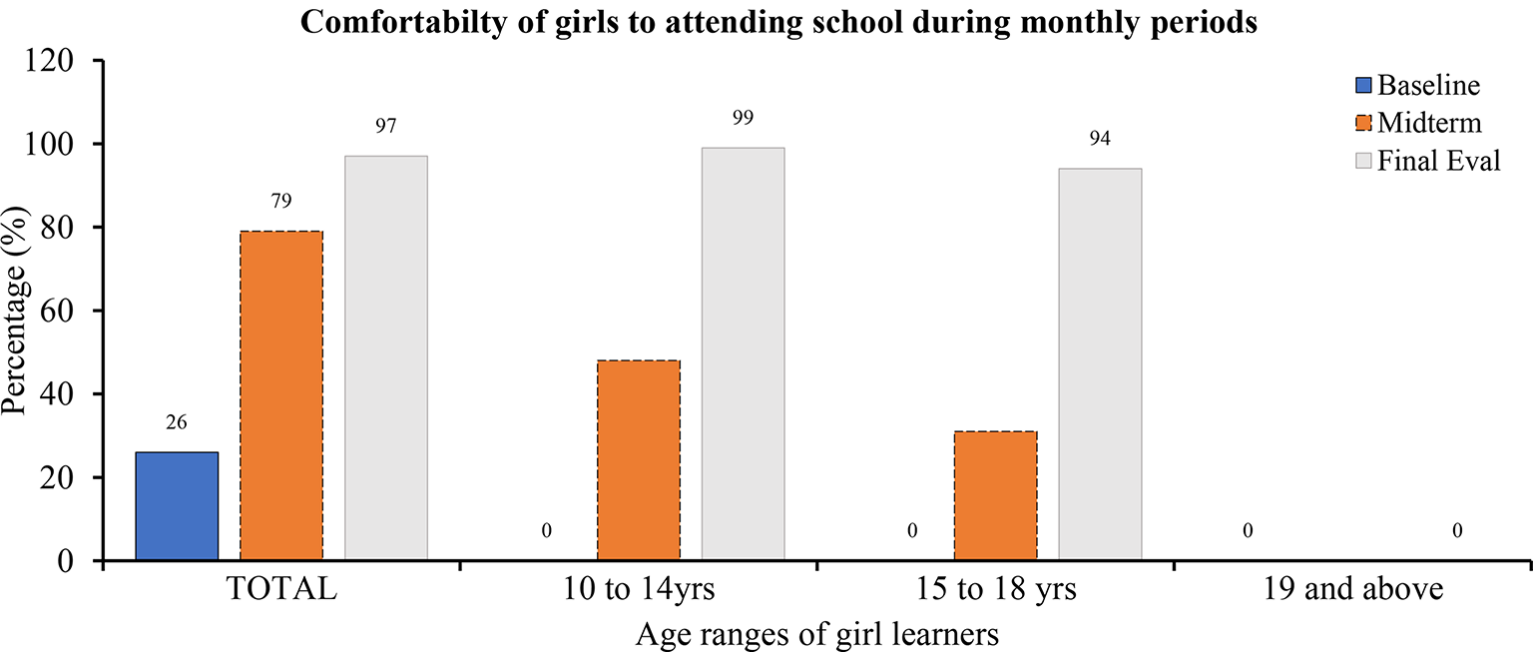
Percentage of girl learners comfortable attending school during menstruation

With the MHM project’s interventions, the majority of girl learners (97%, *n* = 100) became more comfortable attending school during menstruation compared to the pre-project period (Fig. 5). This is an improvement from 26% at baseline to 79.2% during the mid-term evaluation. The girls felt comfortable because they had sanitary pads and menstrual cups that prevented them from messing up during menstruation, hence reducing absenteeism and dropping out. The results from the interviews with girl counselor teachers confirmed that the schools had reusable sanitary pads ready for girls who reached puberty and started menstruation. There was a strong adoption of sanitary products among all age groups, with 99% and 94% coverage for girls aged 10 to 14 and 15 to 18 years, respectively.

#### Use of reusable pads and menstrual cups

**Fig. 6:**
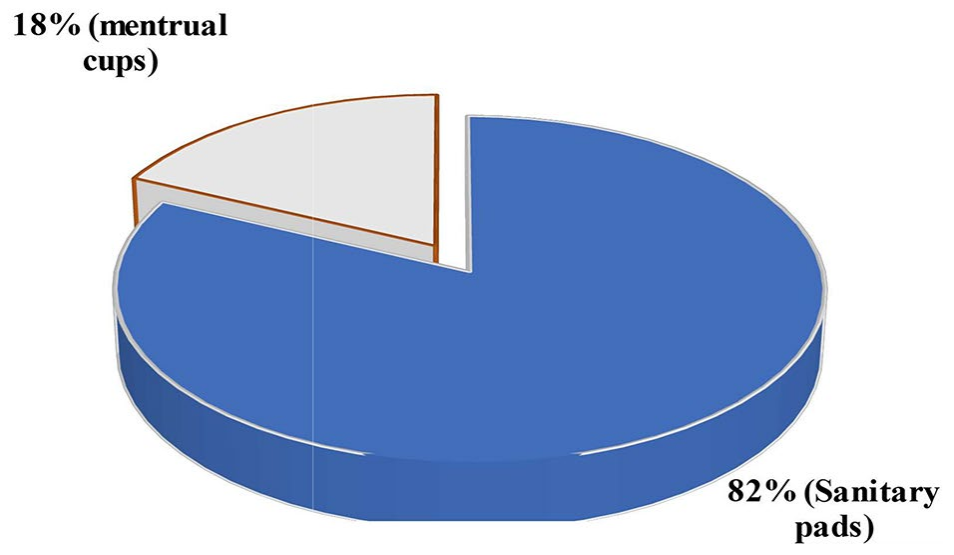
Girls’ preference between reusable sanitary pads and menstrual cups

There was an increase in the number of girls (*n* = 100) using reusable pads (83.2%) compared to menstrual cups (70%). This is because the latter was introduced by the project lately, while the former was fairly being used by some of the students before the project. Furthermore, reusable sanitary pads were fairly cheaper and locally made compared to menstrual cups (Table 3). Analysis by schools showed that Milala (100%) was the best to use menstrual cups compared to Chifeni (32%). This was mainly because the large cup sizes purportedly caused pain to young adolescent girls at Chifeni. Chifeni School had younger adolescent girls who participated in the interventions compared to Milala School. The majority of girls (82%) preferred reusable sanitary pads over menstrual cups (Fig. 6). Very few girls (18%) reported using menstrual cups exclusively for several reasons, such as pain and difficulty to wear. Furthermore, it was revealed that some girls indicated that the cups were large. Through the provision of sewing machines and pad fabrication training, skills and knowledge were gained by women and adolescent girls to produce pads packaged as part of the MHM kit for girls who had just started their menses. Such an initiative was highlighted by both teachers and community members as a success and the project’s sustainability strategy.

**Table 3:** Reasons for girls’ preferences between reusable sanitary pads and menstrual cups

Reusable sanitary pads	
Advantages	Disadvantages
• *Locally available* • *Easily and locally made* • *Easy to use as it doesn’t require any insertion* • *It is cheap* • *Doesn’t cause any pain when using*	• *If not well made, needs to be changed frequently* • *It takes time to dry*
**Menstrual cups**	
• *It is durable and this removes costs that girls and women would incur to access other MHM materials* • *It lasts a long before changing* • *Girls can do a variety of exercises including riding bicycles and other sporting activities without any discomfort* • *It is very easy to clean compared to reusable pads* • *It improves girls’ dignity because it doesn’t add any extra layer of clothing*	• *It is painful to use if not your size* • *Difficult to insert for first-time use* • *Handle with care when emptying to avoid losing it into pit latrines*

The majority of girls (60%, *n* = 145) trained in the fabrication of reusable sanitary pads were actively involved in continuous production, surpassing the project target of 20%. Interestingly, the girls in the age group of 10–14 years scored the highest (99%) in fabricating reusable sanitary pads. Furthermore, the key informant interviews with mother group members, teachers and local leadership showed that mother group members were highly involved in the fabrication of reusable sanitary pads and training other learners, hence representing a great sign of sustainability. During health education talks and parent engagement meetings, men and boys were deliberately included to improve their knowledge and perceptions toward MHM. A total of 458 men and boys (10–14 years, *n* = 187; 15–18 years, *n* = 241 and 19 above, *n* = 30) were reached during the MHM health talks and a quiz on myths and misconceptions. A total of 144 boys out of 180 managed to score 75% above, representing 80%, a percentage slightly higher than the midterm score of 45.2%.

### Improved knowledge and attitudes toward the application of MHM solutions

#### Parents and community members

The percentage of parents that encourage girls to attend primary school even during periods of menstruation was evaluated and disaggregated. This was done to assess their level of knowledge and support given to adolescent girls during the monthly periods. In the final evaluation of the project, the results showed that the majority of the parents (64%) encouraged their daughters to attend school even during monthly periods. This was significantly higher than the midterm (46%) and baseline evaluations (8%). After implementation of the MHM interventions, the majority of the parents and community members (62.3%, *n* = 90) were comfortable with the menstrual products used by their daughters.

#### Boys and men

After MHM interventions and awareness campaigns, many boys gained knowledge about menstruation, especially myths and misconceptions. For example, approximately 80% of the boys trained on MHM were able to score above 75% in the post training quizzes compared to 45% during midterm and 0% during baseline. This is an indication of the absorption of knowledge on MHM. In addition, there were no fathers who felt that discussing issues of menstruation with their children was taboo and against cultural beliefs, with 2.40% at baseline, 1.3% at midterm to 0% at final evaluation. Furthermore, there was a significant difference (*p* < 0.05) in the reaction and attitudes of male parents responding to questions about the menstrual issues of their daughters between the baseline and the final evaluation assessments.

## Discussion

### Availability of wash facilities in schools and surrounding communities

The four schools involved in our study had latrines separate for girls and boys (45% improved, 54% ventilated improved pit latrines – VIPs). However, these schools lacked handwashing facilities and soap for cleaning and handwashing. This is a problem considering that adolescent girls undergoing menstruation require handwashing facilities and soap for sanitary purposes. Correspondingly, two schools namely Chifeni and Kabuthu did not have separate washrooms for changing sanitary materials, hence depriving them of their privacy and dignity during menstruation. Similar results on the lack of washrooms for changing sanitary materials and poor access to clean water, sanitation and hygiene facilities have been reported in Malawi and elsewhere [8, 18]. The lack of latrines both at school and at home has been reported widely in the literature to pose negative impacts on menstrual health for adolescent girls [2, 13, 19]. Furthermore, the absence of proper latrines both in schools and homes has globally been reported to deprive adolescent girls of privacy, safety and dignity to change used menstrual materials within the household in Zambia and Kenya [11, 20]. The decrease in the latrine coverage from 90 to 85.7% at the final evaluation in Kabuthu zone (Chifeni school communities) was attributed to too much rain received in the area, which damaged most of the latrines constructed using unburnt bricks and mud. Although the schools are found within the same geographical area, Milala School communities did not receive too much rain naturally, hence managed to maintain their latrine coverage both at the project midterm and final evaluation (90.9%).

### Absenteeism from school

The reduction in the number of girls failing to attend classes due to menstruation was attributed to the availability of reusable sanitary pads and menstrual cups provided during the MHM project implementation. During the study girls confessed that the pads and the cups gave them freedom and dignity to attend school even during monthly periods. The positive association between retention of girls and the implementation of MHM education talks, parent engagement meetings, and provision of sanitary pads and menstrual cups showed that the MHM interventions were successful. Generally, the tendency of girls to miss classes was attributed to menstrual-related health problems. The lack of medication for managing menstrual-related health problems (such as abdominal cramps, headaches, and backache) for adolescent girls was attributed to a lack of funding and poverty levels.

#### MHM products and training provided in project schools

The current study noted the need to procure age-specific menstrual cups to match the young adolescent age ranges that were prevalent at Chifeni School. The differences in adoption of MHM products among schools were attributed to opposing preferences and attitudes toward menstrual cups, hence a need for more awareness of the utilization of menstrual cups. Varied adoption and use of menstrual cups and reusable pads by adolescent girls have been reported in many countries, including rural Nepal [21], rural western Kenya [18], and in rural schools of Zambia [11]. In this study, at the time of the assessment, the project had made available a total of 3084 reusable pads and 580 cups. A total of 1534 reusable pads were locally produced by mother group members and students, while 1550 pads were procured and distributed to students. Conversely, a total of 580 menstrual cups were distributed to 366 adolescent girls, 23 mother group members, 35 female teachers, and 156 women from the community. Through the provision of reusable sanitary pads and menstrual cups in the four schools, the endline survey results showed a positive adoption and usage of the distributed reusable sanitary pads and menstrual cups. Generally, there was increased participation in reusable pad fabrication by students and mother group members in project schools and the community. The enormous leap in availability and usage of reusable sanitary pads after project implementation was attributed to the provision of reusable sanitary pads and training on the fabrication of pads conducted on 145 adolescent girls (representing 39% of adolescent girls in the Kabuthu education zone) and 30 mother group members from the four primary schools studied. Similar findings on MHM among women and girls in Malawi noted that the use of disposable pads was favored by most girls who participated in the study [8]. Conversely, studies conducted elsewhere showed that rural and poor women and girls resort to using old clothes for menstruation rather than menstrual cups and reusable sanitary pads due to their poor economic status, availability on the local market, cultural acceptability and personal preferences, among others [11, 22–26]. The use of unsuitable and unhygienic menstrual products is reported to increase rates of pelvic pain, lower genital tract infections, and inflammatory conditions, further exacerbating absenteeism [10].

To ensure the sustainability of the MHM interventions in the study area, there is a need to make the menstrual products available on the local market. Furthermore, the project implementers and key stakeholders in the area should consider subsidizing menstrual products so that adolescent girls and women can afford to purchase them. Some scholars and previous researchers have advocated for all-inclusive sanitation and hygiene education in schools [8, 27, 28], and this is the same recommendation in the study area and elsewhere in the country and beyond. Thus, there is a need for comprehensive training on MHM and hygiene education so that all groups (boys, girls, women and men) fully understand and remove discrimination and harmful cultural practices.

#### Knowledge and MHM solutions for community leaders and health workers

The current study showed limited knowledge and understanding of MHM and menstruation by all study participants before MHM education implementation. Similar studies on limited knowledge of MHM by boys and men have been reported in Zambia [11] and in India [29]. However, after MHM project implementation in Kabuthu education zone in Lilongwe, the study demonstrated that MHM education talks and parent engagement meetings played a major role in the improved retention of girls throughout the project lifetime in the Kabuthu education zone. The comfortability of parents and community members with the menstrual products used by their daughters was attributed to increased awareness campaigns and engagement meetings, where information on proper MHM was shared. Such efforts and campaigns changed the attitude of parents toward the use of improved menstrual materials as well as offering MHM support to girls. Furthermore, community leaders and members hailed the MHM project for challenging harmful cultural norms that projected MHM talks to be feminine and taboo. There were 8 community engagement meetings conducted in all the schools (2 per school) where both men (*n* = 240) and women (*n* = 461) participated. The leaflets (400) and posters (32) distributed in the four schools played a crucial role in raising MHM awareness among adolescent girls and community members. Although the project engaged a few healthcare workers (35%, *n* = 20) in facilitating MHM health talks and menstrual cup orientation, the impact was statistically significant (*p* < 0.05). Regarding the perception and knowledge of men and boys in MHM, deliberate efforts made to engage men and boys in addressing menstrual-related challenges such as stigma and bullying adolescent girls at school showed positive results. From the study findings, it is indeed vital that crucial stakeholders such as community members and health workers, parents and community leaders, among others, are included in MHM interventions and programs for sustainability. Such multistakeholder engagement and inclusion also ensure that MHM interventions are monitored and supported even when the project is finished [8].

### Lessons learned and upscaling of the MHM elsewhere

The study has shown that during the implementation of the MHM project, several risks and challenges were encountered. These included cultural resistance to innovation adoption, especially menstrual cups; inflation (adjustment of all the planned activities); COVID-19 lockdowns and restrictions, limited access to MHM products (cups, reusable pad components); cholera outbreaks; and uncertainty of sustainability of the MHM products. To mitigate cultural resistance, the gatekeepers and all key communities and stakeholders were engaged through meetings and the adoption of engagement information, training, and constant impact monitoring of the project in communities surrounding the 4 schools. Several activities were implemented during the same field trip to reduce travel costs and other logistics. During the cholera outbreak, community members surrounding the schools and all members of the school community (learners and teachers) were sensitized to take precautionary measures on how to prevent cholera and to rush to the nearest health facilities in case of cholera cases. On the sustainability of the innovation, the mother group members were sensitized to continue producing the reusable pads and selling the extra ones to the community members so that they would be able to continue buying materials for sewing.

Other lessons learned during the implementation of the MHM in the study area showed that the inclusion and involvement of female teachers and other key stakeholders (mother groups and health officers) during MHM training increased ownership of the project interventions. On the one hand, the presence of female teachers as stakeholders made it easier to manage the delivery of MHM services, including health education. Furthermore, the inclusion of female teachers in the MHM solutions brought girls closer to their teachers and eventually opened up issues of menstruation. On the other hand, the inclusion of boys in the health education talks addressed issues of stigma and discrimination. This allowed for a holistic approach to MHM and helped the boys to better understand issues of MHM, hence support adolescent girls well.

The gender analysis revealed gender gaps in the Kabuthu education zone that exacerbated school absenteeism and dropout among girls. Menstruation was singled out as the main driver of school absenteeism and dropout among adolescent girls. Contributing to challenges of menstruation were cultural and traditional norms related to menstruation, lack of proper and adequate menstrual knowledge and products among girls and communities, lack of adequate female teachers to act as mentors and role models, and stigma and ignorance perpetuating bullying toward adolescent girls. Gratefully, the MHM project addressed these challenges to ensure equitable access to educational learning outcomes for both boys and girls and reduced school absenteeism and dropout related to menstruation among adolescent girls in the Kabuthu education zone. Such lessons learned can be implemented anywhere in Malawi and beyond. To ensure the sustainability and proper promotion of MHM products and hygiene education in the country, the Malawi Government is developing standard protocols, guidelines, and implementation strategies. The ongoing revised National Sanitation and Hygiene Policy (2024) and National Sanitation and Hygiene Strategy (2018–2024) aim to promote user-friendly sanitation and hygiene facilities; establishment of gender equality and social inclusion (GESI) in menstrual health and hygiene management in all sectors; and waste management and MHM in all schools and facilities. The established policy and strategies on sanitation and hygiene in Malawi will ensure that MHM is regulated and provide guidance for decision-making and that menstrual products and services are moderated and accepted for promotion.

## Conclusion and recommendations

This study has confirmed that access to sanitary products in schools and at home is crucial for menstrual hygiene and reducing absenteeism for adolescent girls in Malawi. Some schools did not have separate washrooms for changing sanitary materials for adolescent girls. Furthermore, the use of unburnt bricks and nondurable materials was found to compromise the durability of latrines. The results suggest that there is a need for the construction of girl-friendly and durable WASH facilities and washrooms. Following MHM interventions and implementation, the study has shown a significant reduction in girls who missed classes. The awareness and involvement of boys, men and other community leaders and healthcare workers removed the myths and misconceptions related to menstrual hygiene and the impacts of bullying that result in girls staying away from school or completely dropping out. The study, therefore, calls for comprehensive training on MHM and hygiene education so that all groups (boys, girls, women and men) can remove discrimination and harmful cultural practices. Additionally, the study has shown that key stakeholders such as community members and healthcare workers, parents, mother groups and community leaders must be included to promote the uptake and sustainability of reusable pads and menstrual cups and MHM interventions and programs in general. Further areas of research would include longer-term evaluation of the impact and sustainability of the MHM project to support the observations of this study. Key areas would include assessment of continued access and use of menstrual health products, community involvement and impact on girls’ level of education.

### Electronic supplementary material

Below is the link to the electronic supplementary material.

Supplementary Material 1

## Data Availability

Useful data are presented in Tables and charts in the report. Additional datasets and files for the study are available from the corresponding author upon request.
