# Supplementary material for: Evaluation of knowledge, attitude, practices and effectiveness of menstrual hygiene interventions in rural schools from Lilongwe, Malawi
Source: BMC Public Health. 2024 May 29;24:1435. doi: 10.1186/s12889-024-18940-w (PMC11134943; doi:10.1186/s12889-024-18940-w)
Supplement: Supplementary file 1 — Supplementary Material 1 [file 12889_2024_18940_MOESM1_ESM.docx]

##

## **Appendix 1: Data collection instruments (English)**

1. **GIRLS MHM QUESTIONNAIRE**

**Individual interviews**

| **Introduction** | | |
| --- | --- | --- |
| Freedom to learn for Girls in rural Malawi – Addressing Menstruation Barriers is a one-year research project aimed at reducing school absenteeism and dropout related to menstruation among adolescent girls. The project is being implemented in four Primary schools namely Milala, Chifeni, Kamphelatsoka and Kabuthu primary schools under Kabuthu education zone in Lilongwe rural Malawi. This survey therefore aims at assessing the impact of MHM interventions in the aforementioned primary schools. The interview will last a maximum of 1 hour and participation is voluntary. Please note that there is no correct or wrong answer, your views will be highly appreciated and the answers you will give will be kept confidential. | | |
| **Section A. Participant details** | | |
| A1. Name of the adolescent | A2. Age | A3. Name of the School |
| **Staff details** | | |
| A4. Name of the Interviewer | A5. Time | A6. Date of the Interview |
| **Section B. Menstruation Information** | | |
| **B1. Have you started experiencing menses?** *(If No, skip to B13)*   1. Yes 2. No   **B2. If yes, for how long have you been experiencing menstruation?**  1. Less than 1 year 2. 1-2 years 3. 3-5 years 4. More than 5years  **B3. How many days does your monthly period last?**  1. Less than 3 days 2. 3-5 days 3. More than 5 days  **B4. Do you experience any health-related problems during your periods?** *(If No, skip to B7)*  1. Yes 2. No  **B5. If yes, what exactly do you experience?**  1. Headache 2. Abdominal cramps /pains 3. Vomiting 4. Diarrhea 5. Nausea  6. Dizziness 7. Backache 8. Very tired. 9. Others, specify_______________________________  **B6. How do you manage these health-related problems?**  I. I take medicine 2. I do nothing 3. I use traditional medicine  4. Other specify_______________________________________________________  **B7. Do you feel comfortable discussing your menstruation with others?** *(If No, skip to B9)*  1. Yes 2. No  **B8. If yes, with who?**  1. My mother 2. My father 3. My sisters 4. My relatives 5. My peers  6. Other specify _________________________________________________________  **B9. If no, why?** *(If yes to B8, skip to B10)*  1. I feel shy 2. It is a taboo 3. I get bullied when I discuss with others  4.Lack of confidentiality among those I confide in 5. There is no one to talk to  **B10. Do you miss classes because of menstruation?** *(If No, skip toB13)*   1. Yes 2. No   **B11. If yes, what are the reasons**   1. I get sick 4. I easily mess up and feel shy 2. My parents tell me to be at home 5. Lack of adequate sanitary pads   3. Due to cultural beliefs 6. I get bullied after messing up  7. I am too tired 8. Other specify_______________________________________________  **B12. On average how many days do you stay out of school because of menstruation** *(refer to question B3 for triangulation)*   1. Less than three days 2. 3-5days 3. Above 5 days   **B13. Do you have access to menstrual hygiene management information?** *(If No, skip to C1)*   1. Yes 2. No   **B14. If yes, where do you get such information?**   1. From teachers at school 2. From mother group members 3. From peers   4. From parents/guardians 5. From NGOs 6. From the school library  7. Other, specify ___________________________________________________________  **B15. How would you rate the information you receive on menstrual hygiene management?**   1. Less helpful 3. Inadequate 2. Helpful 4. More helpful   **B16. What challenges do you face when accessing menstrual hygiene management information?**   1. Information not locally available 2. The language of information not user-friendly   3. Am usually busy with other things and have no time to access information  4. Lack of interest in accessing information on MHM  5. Don't know how to find it. 6. Others, specify ________________________________________ | | |
| **Part C. MHM Product Availability** | | |
| **C1. In your previous menstruation, what sanitary materials /products did you use?** *(If No to B1, skip to C3)*  1. Cloth/towel 3. Cotton wool 5. Disposable sanitary pad 7. Under wear alone  2. Reusable sanitary pad 4. Toilet paper 6. Mattress or foam 8. Menstrual cup  9. Others, specify __________________________________________________________  **C2. Where did you get the sanitary materials /products you mentioned?**   1. Bought from the local shop 4. Made by myself 2. Received from CPAR Malawi 5. Given by parent/guardians/relative 3. Received from school 6. Received from mother group   7. Other specify_______________________________________________________________  **C3. Do you know how to sew reusable sanitary pads?** *(If No, skip to E1)*   1. Yes 2. No   **C4. If yes, where did you learn the skills from?**   1. From CPAR Malawi 2. From mother group members 3. From parents/guardian 2. Other specify_______________________________________________________________   **C5. How many reusable sanitary pads do you have?**   1. Less than three 2. 3-5 3. More than 5   **C6. On your heaviest day, how many times do you change sanitary materials?**   1. Once 2. Twice 3. 3 times 4. 4 times 5. More than 5 times   **C7. Where do you most often change your menstrual materials when you are at home?**   1. Bedroom 2. Latrine 3. Bathroom/washing space 4. Bush/field 2. Other specify___________________________________________________________   **C8. Where do you most often change your menstrual materials when you are at school?**   1. Latrine 2. Washroom (separate from latrine) 3. Bush /field   4. Other, specify____________________________________________________________ | | |
| **Part D. Adoption and Effectiveness (This part is only applicable to students who use reusable sanitary pads and menstrual cups)** | | |
| **D1. Do you feel comfortable using reusable sanitary pads? (If No, skip to D3)**   1. Yes 2. No   **D2. If yes, why do you feel comfortable using the reusable sanitary pads? Tick all that apply**   1. It is cheap 4. It is locally found 2. It absorbs the menses perfectly 5. It can be re-used 3. It can last longer without leakage 6. Other, specify____________________________   **D3. If not comfortable, why? *(If Yes to D2, skip to D4). Tick all that apply***   1. It is expensive 3. Not locally available 2. Not hygienic 4. It causes rash/irritation 3. Other specify_________________________________________________________   **D4. Have you ever used menstrual cups? *(If No, skip to D6)***   1. Yes 2. No   **D5. Do you feel comfortable using menstrual cups? (If No, skip to D7)**   1. **Yes 2. No**   **D6. If yes, why do you feel comfortable using a menstrual cup? *Tick all that apply***   1. It can be used for a longer period before changing 3. It is locally found 2. It can be re-used 4. It is easier to wash 3. Others specify ________________________________________________________   **D7. If No to D5, why? *Tick all that apply***   1. It is expensive 3. Not locally available 2. Not hygienic 4. It causes rash/irritation 3. Other specify_________________________________________________________   **D8. In comparison, what is your preferred menstrual product between reusable sanitary pads and menstrual cups,?**   1. Reusable sanitary pads 2. Menstrual cups   **D9. Why is this, your preferred menstrual product?**  ______________________________________________________________________________ | | |
| **Part E. Access to improved sanitation** | | |
| **E1. What kind of toilet facilities do you use at school?**   1. Tradition pit latrine 2. Improved pit latrine 3. Ventilated pit latrine   4. Eco-san compositing latrine 5. Flush toilet 6. Pour flush toilet  7. Other specify __________________________  **E2. In your school, do girls and boys have separate toilet facilities?** *(****If No, skip to E4)***   1. Yes 2. No   **E3. Do the toilets have hand washing facilities?**   1. Hand washing facilities only at girls’ toilets 2. Both boys’ and girls’ toilets have no hand-washing   3. Both boys’ and girls’ toilets have hand-washing facilities  4. Hand washing facilities only at boys’ toilets  **E4. What kind of hand-washing facilities do you use?**   1. No hand washing facilities 3. Running water from tap 5. Tippy tap 2. From basin 4. Pour water over the basin   6. Other (specify)__________________________________________________  **E5. Does your hand-washing facility have water?**   1. Water at all times 2. Water is rarely available 3. No water at all   **E6. Does your hand-washing facility have soap?**   1. Soap at all times 2. Soap is rarely available 3. No soap at all | | |

1. **BOYS GROUP FOCUS GROUP GUIDE**

**Introduction**

Freedom to learn for girls in rural Malawi – Addressing Menstruation Barriers is a one-year research project aimed at reducing school absenteeism and drop out related to menstruation among adolescent girls. The project is being implemented in four Primary schools namely Milala, Chifeni, Kamphelatsoka and Kabuthu primary schools under Kabuthu Education Zone in Lilongwe rural Malawi. This survey therefore aims at assessing the impact of MHM interventions in the aforementioned primary schools. The interview will last a maximum of 1hr 20 minutes and participation is voluntary. Please note that there is no correct or wrong answer, your views will be highly appreciated and the answers you will give will be kept confidential.

**Guiding questions**

**Section 1 focuses on boys’ awareness of the changes that both boys and girls go through during adolescence and their coping mechanisms**

1. *Can you list down changes that take place in boys and during the adolescent stage?*
2. *How do these changes affect boys i.e., relationships with peers, parent’s, education, as well as the relationship with the opposite sex?*
3. *As boys how do you cope with the changes you experience?*
4. *Can you list down changes that take place in girls during the adolescent stage*
5. *How do these changes affect girls i.e., relationships with peers, parent’s, education, as well as relationships with the opposite sex?*
6. *How do girls cope with changes?*

**Section 2 focuses on boys’ knowledge of menstrual hygiene management and their support towards adolescent girls**

1. *What do you understand by the term period/menstruation?*
2. *How do girls get affected by menstruation?*
3. *Where do you access information on menstruation?*
4. *What challenges do you face when accessing information on menstruation?*
5. *Do boys and girls have equal access to information on menstruation?*
6. *In your community, what activities are girls prevented from performing when they are in menses?*
7. *What are the reasons for girls not performing such activities?*
8. *What is the current situation of bullying girls when they soil/stain their clothes with menses?*
9. *What impact does the bullying have on such girls?*
10. **HOUSEHOLD MHM QUESTIONNAIRE**

| **Introduction** | | |
| --- | --- | --- |
| Freedom to learn for girls in rural Malawi – Addressing Menstruation Barriers is a one-year research project aimed at reducing school absenteeism and drop out related to menstruation among adolescent girls. The project is being implemented in four Primary schools namely Milala, Chifeni, Kamphelatsoka and Kabuthu primary schools under Kabuthu Education Zone in Lilongwe rural Malawi. This survey therefore aims at assessing the impact of MHM interventions in the aforementioned primary schools. The interview will last a maximum of 40 minutes and participation is voluntary. Please note that there is no correct or wrong answer, your views will be highly appreciated and the answers you will give will be kept confidential. | | |
| **Section A. Staff details** | | |
| A1. Name of the interviewer |  | A2. Date of interview |
| Participant Details | | |
| A3. Name of the respondent | A4. Age | A5. Name of the nearest school |
| A6. Sex of respondent | A7. T/A | A8. Village |
| A9. District |  |  |
| **Section B. Access to improved sanitation** | | |
| **B1. Do you have a toilet?** *(If no, skip to B3)*   1. Yes 2. No   **B2. If yes, what kind of toilet facility do you use? (please observe)**  1. Traditional Pit latrine 3. Improved pit latrine 5. Flush toilet  2. Ecosan composting latrine 4. Ventilated Improved 6. Latrine Pour flush toilet  7. Other (specify_________________________________________________________________  **B3. Do you have a hand washing facility?** *(If No, skip to C1)*   1. Yes 2. No   **B4. What kind of hand-washing facility do you use?**   1. Running water from tap 3. Tippy tap 2. From basin 4. Pour water over a basin   5. Other (specify) ______________________________________________________________  **B5. Does your hand-washing facility have water? (please observe)**   1. Water at all times 2. Water is rarely available 3. No water at all   **B6. Does your handwashing facility have soap? (please observe)**   1. Soap at all times 2. Soap is rarely available 3. No soap at all | | |
| **Part C. Menstruation Information for Child /ward** | | |
| **C1. Do you know when your daughter is having monthly periods?** *(If No, skip to C3)*   1. Yes 2. No   **C2. If yes, who informs you?**   1. My child 3. My spouse 2. I observe 4. Other, specify ____________________________________________   **C3. If no, why?**   1. It doesn’t concern me 3. It is a taboo 2. I am not told 4. Other, specify ______________________________________   **C4. Do you have time to discuss with your daughter about her menstruation?** *(If No, skip to C6)*   1. Yes 2. No   **C5. If yes to C4, what exactly do you discuss?**   1. Body hygiene cleanliness of sanitary products 3. Proper disposal of sanitary materials 2. Sexual abstinence 4. Sex and STI prevention   **C6. If no C4, why?**   1. It doesn’t concern me 3. It is a taboo and against our culture 2. My daughter does not come to me 4. Other, specify ________________________________   **C7. How do you support your daughter on issues of menstruation?**   1. Provide money to buy sanitary materials 3. Provide materials 5. I do nothing 2. Provide awareness to my daughter 4. Provide sanitary facilities   **C8. Where does your daughter change her sanitary materials?**   1. In her bedroom 2 In the toilet 3. In the field/bush 4. There is no specific place for changing pads   **C9. Do monthly periods make your daughter miss classes?** *(If No, skip to C10)*   1. Yes 2. No   **C10. If Yes, why?**   1. She gets sick 4. She easily messes up and feels shy 2. Her friends bully her for messing up 5. I instruct her to be home 3. Because of cultural beliefs 6. Other, specify ________________________________   **C11. If No to C7, what support do you provide for her not to miss classes?**   1. I encourage her to go to school 3. I provide necessary support like MHM materials and medicine 2. I do nothing 4. Other, specify ____________________________________   **C12. What materials does your daughter use during her menstruation periods?**   1. Cloth/towel 4. Disposable sanitary pad 7. Underwear alone 2. Reusable sanitary pad 5. Toilet paper 8. Menstrual cups 3. Cotton wool 6. Mattress or foam 9. Other, specify __________________   **C13. Are you happy with the materials she uses?**   1. Yes 2. No   **C14. If no C11, what materials would you feel comfortable for her?**   1. Disposable pads 2. Reusable pads (local cloth) 3. Reusable pads (manufactured) 4. Menstrual cups | | |

1. **MOTHER GROUP FOCUS GROUP DISCUSSION GUIDE**

**Introduction**

Freedom to learn for girls in rural Malawi – Addressing Menstruation Barriers is a one-year research project aimed at reducing school absenteeism and dropout related to menstruation among adolescent girls. The project is being implemented in four Primary schools namely Milala, Chifeni, Kamphelatsoka, and Kabuthu primary schools under Kabuthu Education Zone in Lilongwe rural Malawi. This survey therefore aims at assessing the impact of MHM interventions in the aforementioned primary schools. The interview will last a maximum of 1hr 20 minutes and participation is voluntary. Please note that there is no correct or wrong answer, your views will be highly appreciated and the answers you give will be kept confidential.

**Guiding questions**

**Section 1 focuses on understanding the work of Mother groups within the schools and their relationships with other stakeholders.**

1. *For how long have you been operating as a mother group member at this school?*
2. *As a mother group, what activities do you carry out at this school?*
3. *What support do you receive from the community when carrying out your work?*

**Section 2 focuses on understanding mother groups’ knowledge of girls’ menstrual hygiene management and their involvement in handling such issues**

1. *As a mother group, what activities do you carry out about adolescence and menstrual hygiene management?*
2. *What menstrual-related challenges do girls face in your school?*
3. *How do you resolve such challenges?*
4. *What other structures in your school support girls in menstrual hygiene management?*
5. *What kind of support do such structures render to girls in the school?*
6. *What impact do such activities have on girl education? (Support includes from mother groups and other structures)*
7. *What challenges do the girls face when accessing sanitary materials?*
8. *How (where) do girls in your school access messages/information on menstruation?*
9. *What challenges do the girls face when accessing information on menstrual hygiene management?*
10. *How do you resolve such challenges?*
